# Dr. Trotter, on Gastrodynia

**Published:** 1805-02-01

**Authors:** T. Trotter

**Affiliations:** Newcastle


### *To the Editors of the Medical and Phyfical Journal.*

GENTLEMEN,

A Few reflections still remain to be offered on the Case of Gastrodynia, published in your 68th Number, according to promise. You have seen the manner in which I found myself compelled, from honourable motives, to withdraw my attendance. It appeared to me not consistent with my ideas of propriety, to receive emolument where I could no longer be useful: and it was not compatible with professional integrity, to be a passive spectator of a practice, which my colleague, in this case, could neither explain on his own principles, nor defend by those of any other authority. Some good probably arose from my short note to the husband; as it put him on his guard, and perhaps, prevented the threatened exhibition of *jalap and calomel!* For, according to a rule of comparison, If a saline enema, in fourteen hours, could procure seven dejections; what number must follow such a cathartic as jalap and calomel?

As soon as I was informed of the lady having dismissed her physician, which might be eight weeks from my retirement, I sent the case to her husband, that he might know the particulars in which I differed from Dr. Clark; and with the permission to copy it, if he chose to submit it to any London physician, who might be acquainted with the lady's constitution. Had this been complied with I should have abided by the decision. The difference of opinion between Dr. C. and myself, like all similar disputes, was now animadverted upon in some circles, with an asperity bordering on rancour. Not being an entire stranger stranger in Newcastle, my cause was not left quite undefended; my few friends justified me with becoming firmness and spirit. It was however dogmatically decided, that "*it was presumption in Dr. Trotter to give an opinion on a disease different from Dr. Clark.*" Not being satisfied with the oracular authority of the sapient Pythias, who thus assumed the office of Priestesses to the Delphic god, I determined to send the case to your Journal, that professional men might judge my conduct; the only tribunal where its merits ought to be tried, as being alone competent to allow for the extraordinary situation in which I have been placed.

In thus laying the case before the public, I chiefly plead, *self-defence*, but, in another view, it is highly instructing; and it were well if all such dissentions were submitted to the public eye. Nay, had it not been for the liberal spirit of the MEDICAL JOURNAL, my reputation as a professional man must have been in danger. That there are many circumstances in medical consultation that may be unfit for the public eye, I readily concede: but that a consultation of physicians is to be considered as a Divan, or an Inquisition, where the lives of human beings are to be disposed of without the knowledge of society, has never yet been decreed by medical jurisprudence. The publication of cases, and animadversions upon them, have been admitted in all ages and times: it has prevented the ignorant and illiberal mind from taking shelter under the recovery of the patient, as daily done by the Quack and the Charlatan: it has given spirit to discussion; and collisions of sentiments and opinions, have in a great measure brought the healing art to its present perfection. A large proportion of medical gentlemen, in the present day, are employed in the Navy and Army of the Country: these gentlemen, in their turn, have all to retire from the public service, on a very slender allowance; or, in the emphatic language of Counsellor Erskine, "shivering and poor," as myself; and have only to look to the fruits of an honest industry, in the private exercise of medicine, for the support of their old age. But if they are to be brow-beat and worried, in their duty, by every affluent brother, they may be deprived of even the privileges of a virtuous poverty. It does not much redound to the honour of the age, that there are men among us, who, assuming all the airs of a Radcliffe without the talents, are invidious and jealous inquisitors of the practice of others, and surly dictators in their own; whose

 will will is their law: *Stat pro ratione voluntas;* and "*Bear, like the Turk, no brother near the throne.*"

In being associated with Dr. C. in this case, I met him with all the candour and openness of friendship, and with the respect due to a senior. I venerate age; but that veneration is not to be carried to the length of sacrificing independence of opinion. My sentiments in opposition to his, were delivered without the least tincture of resentment; and they have been published without the smallest feeling of revenge. He has it in his power to reply: but by silence it is not given him to treat me with contempt. From being his equal in professional rank, I am secured against that; and as his superior, in those gradations of study and education that lead to the fair and regular acquirement of medical honours, I am not afraid of being obscured by the bulk of his shadow. The case, as it stands on record, precludes the possibility of credit being derived from recovery; for recovery, after such treatment, only proves that the powers of Nature are sometimes paramount to opposing the grossest violation of medical science. But what is to repay the patient for the superadded pains which she endured? Or what can recompence a mother's affections for being deprived of that first pleasure, *the office of suckling her infant?*

The predicament in which I stand, with respect to Dr. Clark, is not singular; it is nearly the same with that of all the other physicians here, except one, who is his only consultant, and the occasional attendant on his patients. In the only other case, in which I have been joined with him, I also differed widely in opinion. I have, since then, followed him in another; and must confess, that his treatment was equally unaccountable. A man, that in his medical discipline, dissents so far from the established maxims of the profession, must be constantly exposed to feuds and animosities among his brethreu. When a physician in consultation appeals to his own experience, it has been usual for him to give reasons for preferring particular modes of his practice. In the case which I have given, Dr. C. was either unable to do this; or, what is less to be justified, he did not chuse to do it; a species of dictatorship which the sciences do not acknowledge. Nevertheless, I shall not be afraid of meeting Dr. C. in consultation again: I will not abridge my humble sphere of being useful to a human being in sickness, from the terror of any man alive: and if I should find occasion for dissenting dissenting from him, I shall not deviate from my former moderation.

Having encroached thus far on your patience, I shall defer my comments on the case to another Journal. This affords me the opportunity of calling on Dr. Clark to defend his practice by matter of fact, and open discussion, and not by assertions in the gross, as he has done hitherto. He cannot complain that I have indicted him before an improper tribunal, when all the talents and learning of the profession are to be umpires between us.

I am, &c.

